# Automatic fault detection of sensors in leather cutting control system under GWO-SVM algorithm

**DOI:** 10.1371/journal.pone.0248515

**Published:** 2021-03-24

**Authors:** Ke Luo, Yingying Jiao

**Affiliations:** School of Information and Electrical Engineering, Shandong Jianzhu University, Jinan, Shandong Province, China; Torrens University Australia, AUSTRALIA

## Abstract

The purposes are to meet the individual needs of leather production, improve the efficiency of leather cutting, and increase the product’s competitiveness. According to the existing problems in current leather cutting systems, a Fault Diagnosis (FD) method combining Convolutional Neural Network (CNN) and the Support Vector Machine (SVM) of Gray Wolf Optimizer (GWO) is proposed. This method first converts the original signal into a scale spectrogram and then selects the pre-trained CNN model, AlexNet, to extract the signal scale spectrogram’s features. Next, the Principal Component Analysis (PCA) reduces the obtained feature’s dimensionality. Finally, the normalized data are input into GWO’s SVM classifier to diagnose the bearing’s faults. Results demonstrate that the proposed model has higher cutting accuracy than the latest fault detection models. After model optimization, when *c* is 25 and *g* is 0.2, the model accuracy can reach 99.24%, an increase of 66.96% compared with traditional fault detection models. The research results can provide ideas and practical references for improving leather cutting enterprises’ process flow.

## 1. Introduction

The textile industry is vital in the early stage of China’s Reform and Opening-up, which has boosted China’s economic growth directly [1]. The leather industry occupies the majority of the textile industry. Traditionally, leather is cut by hand. However, manufacturers have begun to utilize high-efficiency and high-performance leather cutting devices due to the increasing demand for leather products [2]. Most manufacturers employ the high-frequency vibration Computer Numerical Control (CNC) cutting machine because of advantages such as fast cutting speed, good cutting quality, and high utilization [3]. However, large CNC cutting machines are imported. Maintaining these machines is troublesome because of intellectual property protection and the high costs of after-sales services, which dramatically limits industrial development [4]. Researches on the control system of CNC cutting machines in China are backward. Only some Chinese enterprises produce clothing-cutting machines; most Chinese factories utilize relay-contactor control technology, but the cutting accuracy is low. Because the thickness of the cut fabric is minimal, the relay-contactor control technology can cut clothing samples only, which is challenging to meet large clothing factories’ developmental needs [5]. As a traditional leather cutting device, the cutting machine is vital in improving leather cutting accuracy and tire production efficiency [6]. Therefore, constructing a fault detection model for the original device is urgent [7]. Fault Diagnosis (FD) technology is based on reliability theory, system theory, cybernetics, and information theory. FD is gradually formed by combining modern testing instruments and computers with various diagnostic objects [8]. FD technology can find various abnormal situations punctually and correctly, thereby preventing faults or minimizing the loss of faults in time [9]. Therefore, constructing a fault detection model for cutting control systems in the leather industry is essential to improve industrial efficiency.

Many scholars have researched automatic fault detection algorithms for machines. Ellefsen et al. (2019) proposed a fault detection algorithm for maritime components based on unsupervised reconstruction. They found that this algorithm was very suitable for end-to-end system solutions in the future [10]. Yılmaz and Bayrak (2019) proposed a new fault detection method based on non-wavelet transform to overcome wavelet transform limitations in real-time applications. This method had a wide application range and could detect faults quickly, which was reliable for the microgrid [11]. Okaro et al. (2019) proposed a machine learning algorithm for the automatic detection of product failures. This method was semi-supervised and could use data from builds where the components had been certified and builds where the component quality was unknown [12]. Ellefsen et al. (2020) proposed a fault-type independent spectral anomaly detection method for autonomous ferries, achieved 97.66% accuracy in the final test [13]. Brigham et al. (2020) proposed simplified automatic fault detection in wind turbine induction generators, with the rotor’s electrical asymmetry. This method was robust in variable speed. It also showed good versatility when it detected failures at speeds and conditions that did not occur during training [14]. To solve equipment failure, De Martini and Facchinetti (2020) proposed an electromechanical system framework based on a fuzzy inference system. This method and its specific conditions for electric vehicles had high computational performance and accuracy [15]. Therefore, deep learning algorithms and AI optimization are often utilized for fault detection. Nevertheless, different algorithms vary significantly.

Regardless of the above problems, the primary contributions are: (1) the Support Vector Machine (SVM) in deep neural networks is introduced into the leather cutting control system, which effectively solves the difficulty of determining the neural network, the local minima, the over-learning, and the under-learning. (2) The Gray Wolf Optimizer (GWO) is introduced to improve the algorithm accuracy. The accuracy of model clipping is improved further through parameter optimization. (3) Although many works have also applied the above two algorithms, the fusion algorithm’s efficiency is not improved. The research results can provide a practical basis for the technological development of the leather industry.

## 2. Related works

### 2.1 Principles of cutting control system

**Fig 1:**
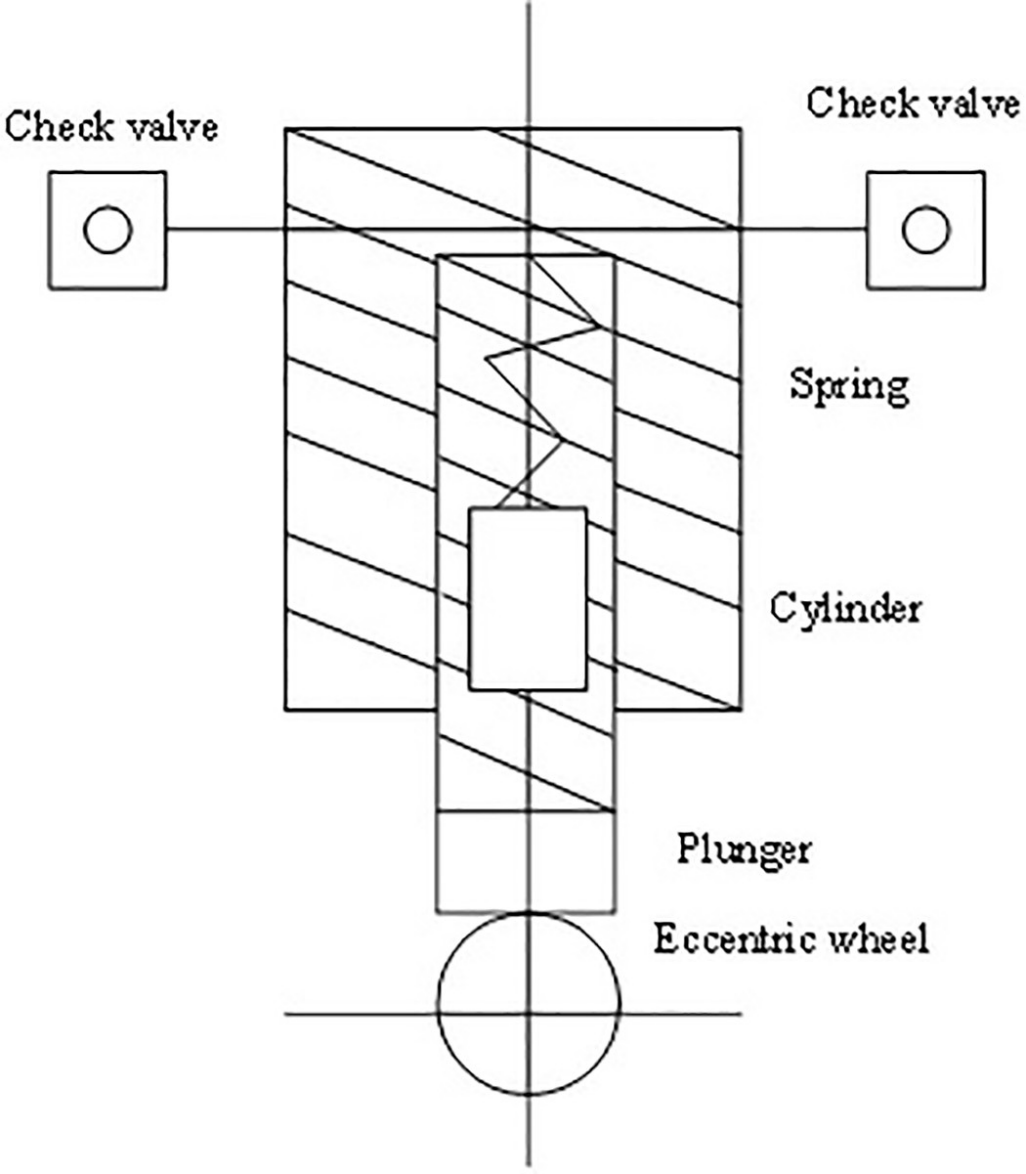
Hydraulic control system of cutting machine.

The leather cutting system works through hydraulic transmission and control. Notably, its structure is shown in Fig 1. The cutting control system uses hydraulic oil as the primary medium. The hydraulic pump generates tremendous pressure; then, the power components convert mechanical energy into hydraulic energy. The logical hydraulic control system employs hydraulic energy to control the angle and accuracy of leather cutting. Finally, hydraulic oil pressure is converted into mechanical energy via a cylinder or motor, thereby driving the mechanical load blade to move linearly or rotationally; consequently, the leather is cut [16].

The cutting machine’s hydraulic control system is the core to ensure cutting accuracy and efficiency, including power components, control components, and executive components. The principle of this system is similar to that of hydraulic transmission. However, the core control system will have more feedback devices, and the actual situation can be fed back to the computer while the hydraulic pump and its motor are cutting [17]. Principal parameters include actuator output, such as the displacement, speed, and pressure of the tire or leather. These parameters are compared with the input before cutting. The deviation between the input and the output is kept constant according to relevant departments’ requirements, thereby meeting the accuracy of leather cutting. Here, the core is the series feedback of multiple sensors, critical for leather cutting [18].

### 2.2 Research progress of FD

With the continuous development of signal collection technology, data processing technology, and computer technology, scholars worldwide have obtained many theoretical results in FD, and new diagnostic methods have also been continuously developed and improved, which greatly improved the reliability of fault monitoring and diagnosis. The United States has established a working group for mechanical fault monitoring and preventive diagnosis. This group is engaged in research on aviation equipment failure analysis and prediction [19]. In the meantime, mechanical FD technology has received much attention in European countries. The United Kingdom has established a machine health center engaged in mechanical FD research [20]. The Danish B&K Company has developed advanced sensor manufacturing technology. With the development of sensor technology, some scholars begin to use various sensors to collect signals under the working state of machinery and analyze the signals to evaluate the status of rolling machinery [21]. With the application of Fourier transform technology in signal processing, researchers begin to introduce spectrum analysis technology into the FD of motor rolling machinery, such as comparing the characteristic frequency of the vibration signal collected by the acceleration sensor with the characteristic frequency obtained by theoretical calculation or spectrum analyzer, thereby determining whether the working state of the rolling machinery has changed [22]. The “resonance demodulation” technology can separate fault signals and effectively determine the location and severity of mechanical faults [23]. With the development of computer network technology, researchers focus on developing online monitoring systems and expert systems for rolling machinery. Although expert systems can solve remote monitoring problems very well, it cannot extract fault features, or the extracted features are incomplete. The diagnostic accuracy of the method is not high.

A series of theories and results have been accomplished in the research of the FD algorithm. Hsu and Liu (2020) proposed a Convolutional Neural Network (CNN) intelligent diagnosis algorithm, which could automatically extract the mechanical fault features and recognize the faults. The feasibility of this method was proved through experimental simulation [24]. Amirat et al. (2020) put forward a method based on variable modal decomposition combined with the optimized SVM network for joint FD [25]. Qu et al. (2017) combined sparse expression technology and used its advantages in signal processing to extract features and identify faults of rolling machinery fault signals, achieving better diagnostic results [26]. Xu et al. (2017) designed a mechanical FD method based on LMD and morphological filtering. The reliability and feasibility of this method were verified by building a railway freight car wheel-to-rolling mechanical test system and analyzing typical mechanical failure signals [27]. Yu et al. (2016) designed a scheme of a rolling mechanical FD system based on LabVIEW, which analyzed and processed the signals under the diagnosis platform of LabVIEW. The feasibility of the scheme was verified through simulation test results [28]. Hong et al. (2017) proposed an early FD method for wind turbine machinery based on MCKD-EMD. The maximum correlation kurtosis deconvolution could highlight the fault shock pulse signal covered by noise in the mechanical vibration signal. The combination of MCKD and EMD was applied to early mechanical FD [29]. Zhu et al. (2019) proposed rolling machinery fault detection and diagnosis based on compound multi-scale fuzzy entropy and integrated SVM [30]. Ma et al. (2018) put forward a mechanical FD method based on wavelet packet decomposition and Principal Component Analysis (PCA) [31]. Deng et al. (2018) combined empirical mode decomposition with independent component analysis. They proposed an FD method based on empirical mode decomposition and independent component, which was successfully applied to mechanical FD [32]. Hu et al. (2019) proposed a rolling mechanical FD method based on feature extraction of compressed information. Apparently, with the development of technology, the methods of FD have also become diversified. However, these studies mostly stay in the theoretical stage and use less in actual production.

### 2.3 Fault detection model

**Fig 2:**
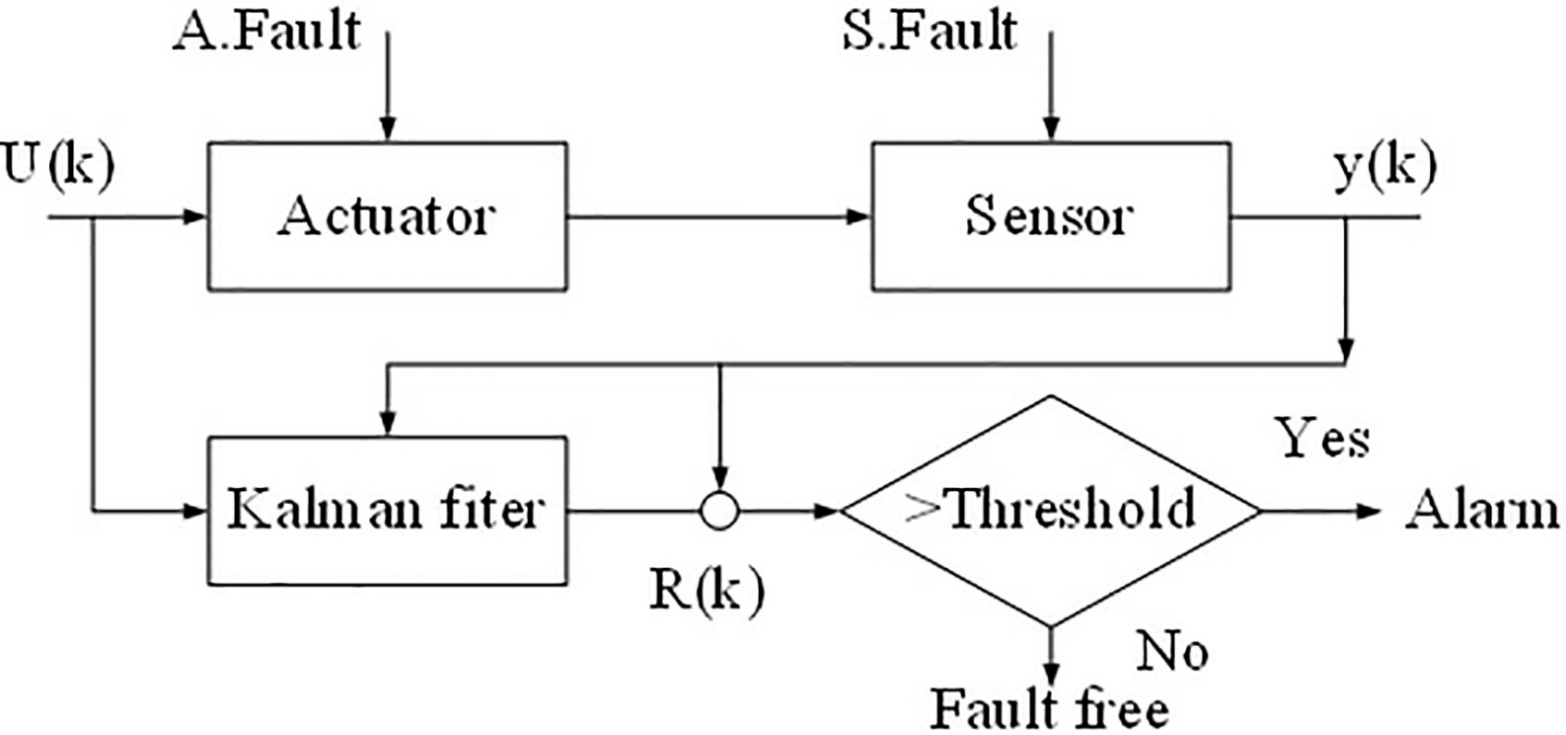
Fault detection model.

The Programmable Logic Controller (PLC) processor, data collection, data storage, and data communication need to be placed on the same data processing platform for fault detection inside the leather cutting system. Multiple sensors must monitor the overall circuit jointly since the cutting system’s internal circuit and the running process are complicated. Fig 2 illustrates the internal fault detection system of the cutting machine. First, the system transmits data to the sensor through the operation. The sensor uploads the running data to the central processing system in time, and the central processing system is coordinated and dispatched via unified coordination, which is convenient for the operator to analyze and judge the overall operation of the cutting machine, evaluate the possible problems of the cutting machine, and thereby arranging the following production.

FD of the cutting system is the key to device operation. On the one hand, nature, degree, class, location, cause, and development trend of the fault can be determined, thereby providing an accurate reference for the following forecasting, control, adjustment, and maintenance. On the other hand, experiences can be accumulated for future FD of the cutting system, and appropriate solutions can be chosen for different fault types and degrees. However, the cutting control system’s current fault detection has problems, such as low detection accuracy and low recognition efficiency. Most of the faults are judged based on human experience, which significantly limits fault detection technology development. Therefore, the way to learn and judge fault detection is fundamental.

Factories are becoming increasingly intelligent and generating loads of process data with the rapid development of sensor technology, data storage and the internet. Data analysis needs for large amounts of data arise at the historic moment, and data-based machine learning technology can effectively improve FD. Commonly used fault detection technologies include Bayesian network, Artificial Neural Network (ANN), SVM, and Hidden Markov Model (HMM). Bayesian network is a commonly used machine learning technique for fault detection. It is a white box model because the graphical representation allows users to intuitively and easily understand the interaction between model variables. This characteristic is beneficial for modeling uncertainties and makes it easier for the model to use data from multiple sources. ANN is a non-parametric machine learning algorithm inspired by the functions of the human central nervous system. Its adaptive feature provides a robust modeling function, which is suitable for the nonlinear relationship between features. The similarity between ANN and a biological neural network is that both can calculate the various parts of the function collectively and in parallel, without the necessity of describing each unit’s specific tasks. ANN’s non-parametric nature and the ability to model nonlinear and complicated problems with high precision make it applicable in FD problems. ANN is easy to initialize because it does not require specifying the network structure. SVM uses different kernel functions (such as radial basis functions) to find a hyperplane that can best separate the data, with good classification performance when used with a small training set. SVM is an excellent technique for modeling linear and nonlinear relationships. Compared with other non-parametric techniques, its calculation time is relatively short. The availability of large training datasets is a challenge in machine learning. However, even in the case of limited training data, SVM has good results. HMM is an extension of the Markov chain model, which estimates the probability distribution of state transition and measurement output in a dynamic process, assuming that the process’s state is unobservable. HMM is a probabilistic model and is excellent in terms of unobservable states (such as chemical processes or the health of equipment) during the modeling process; hence, it is very suitable for FD.

### 2.4 GWO for solving engineering problems

There are many reports about GWO solving engineering problems. Fu et al. (2019) proposed a novel method for rotating machinery FD improved by blind parameter identification of MAR model and abrupt hybrid GWO. Signals collected from different fault types were divided into intrinsic mode function datasets through variational mode decomposition, and multiple autoregressive models of all IMFs were established. Afterward, key features were extracted through decomposition and recognition models and PCA. The results proved the effectiveness and superiority of this method [33]. To improve the accuracy and recognition efficiency of bearing DF, Huang et al. (2019) put forward an FD method based on improved GWO and SVM, where SVM was optimized by GWO to obtain the most suitable parameters of the new diagnostic model. Ultimately, this model improved the problem that the algorithm was easy to fall into the local optimum [34]. Li et al. (2020) proposed an optimized binary SVM classifier based on GWO to identify the pantograph arc. Then, the contribution rate of each feature value was calculated according to the current data state obtained from the pantograph experiment. The feature value data with a high contribution rate functioned on the training samples for learning and recognition via the classifier optimized by GWO. The results showed that GWO could quickly and accurately identify the pantograph arc. The obtained classification model was more accurate than the commonly used Genetic Algorithm (GA) and Particle Swarm Optimization (PSO) algorithm [35]. Almomani (2020) proposed a feature selection model for NIDS. The model was based on PSO, GWO, Firefly Algorithm (FFA), and GA, aiming to improve NIDS performance. It used GA, PSO, GWO, and FFA to deploy wrapper-based methods for selection. Anaconda Python Open Source was used to implement its functions. The proposed feature selection model could effectively identify and discover computer network attacks [36]. The above works prove that GWO has been widely applied to solve the engineering problems, especially in fault identification and processing.

## 3. Model construction

### 3.1 Support vector machine

**Fig 3:**
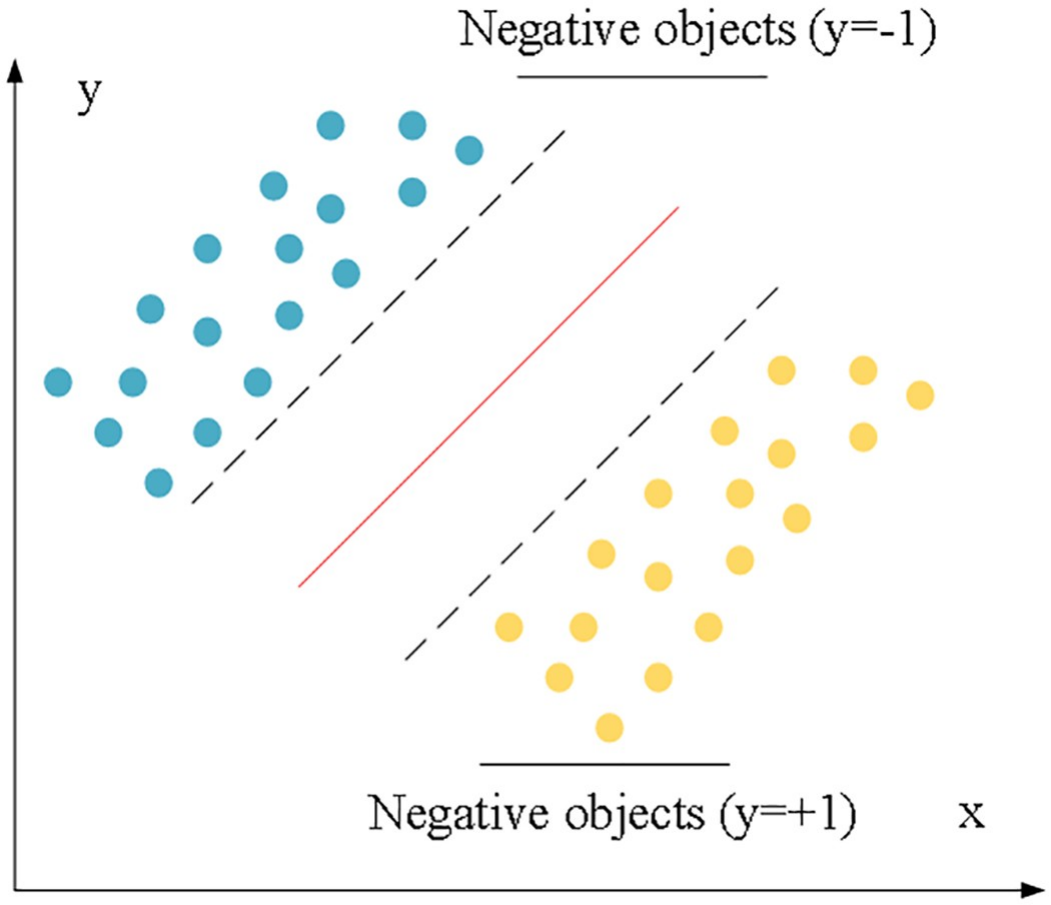
Schematic diagram of SVM principles.

SVM is a supervised learning model for analyzing data in classification and regression analysis in machine learning. SVM is based on the theory of statistical learning knowledge, which can analyze data, classify the samples, and process the nonlinear problems effectively [37]. Essentially, SVM aims to find an optimal classification hyperplane from multiple classification planes. Specifically, the structure of the SVM is shown in Fig 3. The two dashed lines represent the plane supported by the points closest to the optimal classification surface in the samples of the two classes, the red line represents the optimal classification hyperplane, and the data samples on these dashed lines are support vectors [38].

**Fig 4:**
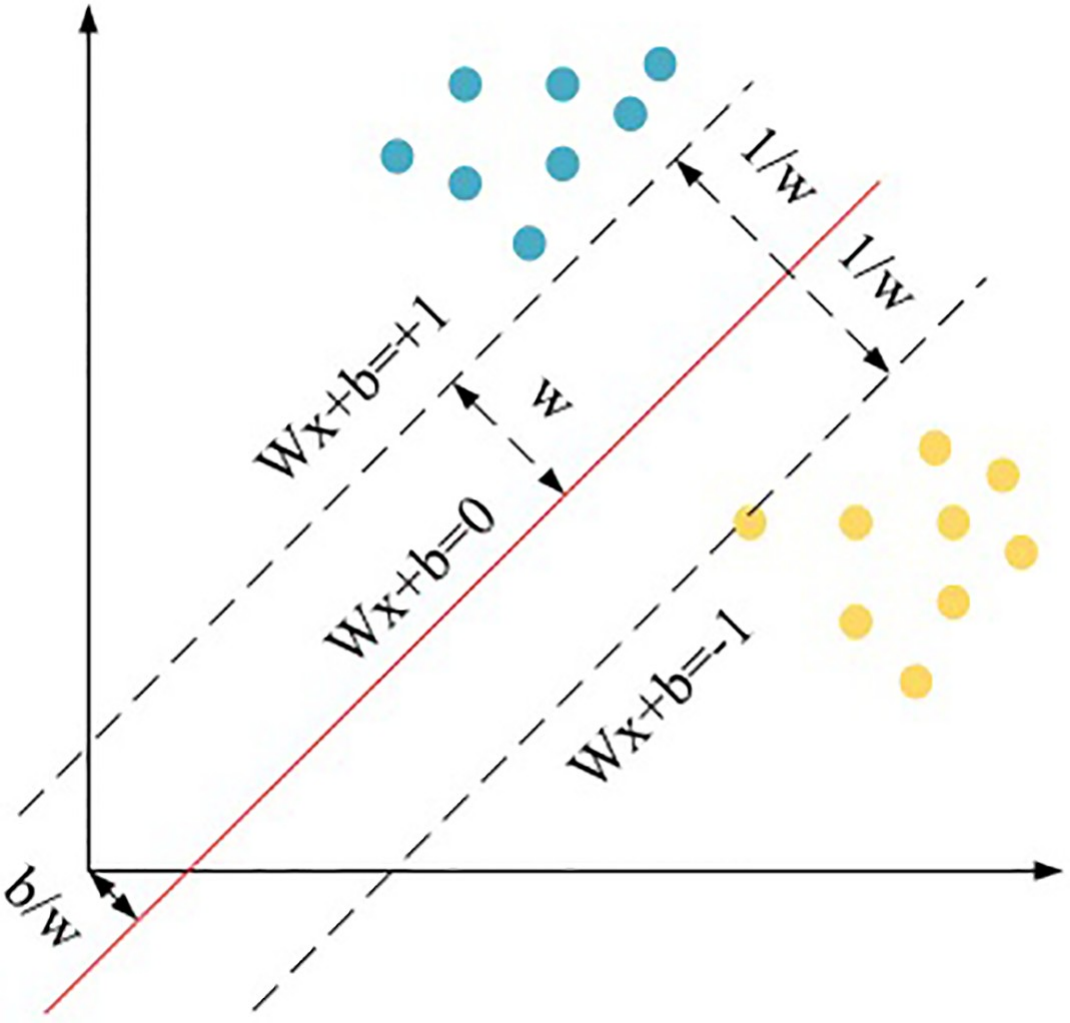
Mathematical expression of SVM.

In Fig 4, the yellow and blue symbols represent two different types of samples, provided that the two types of samples are linearly separable. The sample set to be classified is {*x*_*i*_, *y*_*i*_}, *i* = 1, 2, …*n*, *y*_*i*_ ∈ {−1, +1}, *x*_*i*_ ∈ *R*^*d*^; *y*_*i*_ is the class label number of sample *x*_*i*_, and the value is [-1, +1]; the sample is a *d*-dimensional vector. SVM algorithm aims to find a straight line to separate the two parts and maximize the shortest distance between the plane and the two samples. The function of the hyperplane is denoted as *f (x)*, and it is calculated as:

$$f(x)=\omega^{T}x+b$$ (1)

In (1), *ω* represents the effective distance from the hyperplane to the sample, *T* is the coordinate of the support vector sample point, and *b* is the intercept. If *f (x)* is greater than 0, the sample point is above the hyperplane, indicating that the sample point is positive, and the label value is +1; if *f (x)* is less than 0, the sample point is below the hyperplane, indicating that the sample point is negative, and the label value is -1; if *f (x)* is 0, the sample point is above the hyperplane. The geometric distance between the sample point and the hyperplane is calculated as follows.

$$\tau =\frac{\left|\omega^{T}x+b\right|}{\left\|\omega \right\|}$$ (2)

In (2), *τ* is the geometric distance of each sample point on the hyperplane. The algorithm aims to find the optimal hyperplane. Hence, the distance between the nearest sample of the dividing line and the dividing line should be as far as possible. If the sample is *(x*_*k*_, *y*_*k*_*)*, the objective function with constraints and optimization can be obtained.

$$\frac{y_{i}(\omega^{T}x_{i}+b)}{\left\|\omega \right\|}\geq \frac{y_{k}(\omega^{T}x_{k}+b)}{\left\|\omega \right\|},i=1,\ldots n$$ (3)

$$\text{max}\;\omega ,b\frac{y_{k}(\omega^{T}x_{k}+b)}{\left\|\omega \right\|}$$ (4)

In (3) and (4), *y*_*i*_ is the distance from point *i* to the *y*-axis, and *y*_*k*_ is the distance from point *k* to the *y*-axis. If the nearest point on the hyperplane satisfies *|f(x)| = 1*, the mean distance to the hyperplane will be τ = 1/||*ω*||; hence, the classification distance between the two class samples is τ = 2/||*ω*||, and the exact classification function values are:

$$\{\begin{array}{ll} \omega^{T}x_{k}+b\geq +1, & y=+1\\ \omega^{T}x_{k}+b\geq -1, & y=-1 \end{array}$$ (5)

The expression of learning objective is changed into a mathematical form:

$$\text{max}\frac{1}{\left\|\omega \right\|}$$ (6)

$$s.t.y_{i}(\omega^{T}x_{i}+b)\geq 1,i=1,2\ldots n$$ (7)

In (6) and (7), *s*.*t*. represents the constraint condition. If the optimal plane satisfies *y*_*i*_(*ω*^*T*^ *x*_*i*_ + *b*) ≥ 1, *i* = 1, 2…*n*, the objective function 1/||*ω*|| obtains the maximum value equivalent to the minimum value. Then, the original analysis process can be changed into:

$$\text{min}\frac{1}{2}\|\omega {\|}^{2}$$ (8)

$$s.t.y_{i}(\omega^{T}x_{i}+b)\geq 1,i=1,2\ldots n$$ (9)

The above equations show that under the constraint of inequality, the original problem can be transformed into a dual problem using Lagrange through mathematical analysis; that is, into an equality constraint problem. After the Lagrangian transformation changes it into a dual problem, the optimal ||*ω*|| is searched. Once the constraints are satisfied, the dual problem becomes a set of *α* to maximize the objective function. Hence, the original problem is transformed from an inequality problem to an equality problem. If a set of new samples needs to be predicted for class labels, the following equations are applied.

$$f(x)=\text{sgn}(\sum_{i=1}^{n}\alpha^{*}{}_{i}x_{i}yi(x\cdot x_{i})+b^{*})$$ (10)

$$\omega^{*}=\sum_{i=1}^{n}\alpha^{*}{}_{i}x_{i}y_{i},b^{*}=-\frac{1}{2}\omega^{*}(x_{i}+y_{i})$$ (11)

In actual situations, a penalty factor parameter *c* is introduced for nonlinear problems, whose expression is:

$$\text{min}\frac{\|\omega {\|}^{2}}{2}+C\overset{n}{\underset{i=1}{\sum }}\zeta_{i}$$ (12)

$$s.t.\{\begin{array}{l} y_{i}(\omega \cdot {\text{x}}_{\text{i}}+\text{b})\geq 1-\zeta_{i}\\ \zeta_{i}>0 \end{array},i=1,2,\ldots n$$ (13)

In (12) and (13), *ζ*_*i*_ represents the slack variable, which represents the allowable data point to deviate from the interval, $\sum_{i=1}^{n}\zeta_{i}$ represents the overall possibility of training errors. Parameter *c* can control the tolerance of the sample training credibility; the larger the *c*, the greater the importance of the sample. The feature space product can be calculated directly by introducing the kernel function and using the original space’s input data. The data are then mapped to the high dimension through the Lagrangian transformation and the kernel function and classified effectively by adjusting the penalty factor parameters. Finally, the classification results are obtained. K represents the coefficient, and the specific calculation is as follows:

$$f(x)=\text{sgn}(\overset{n}{\underset{i=1}{\sum }}\alpha^{*}{}_{i}y_{i}K(x_{i},y_{i})+b^{*})$$ (14)

### 3.2 Gray wolf optimizer

**Fig 5:**
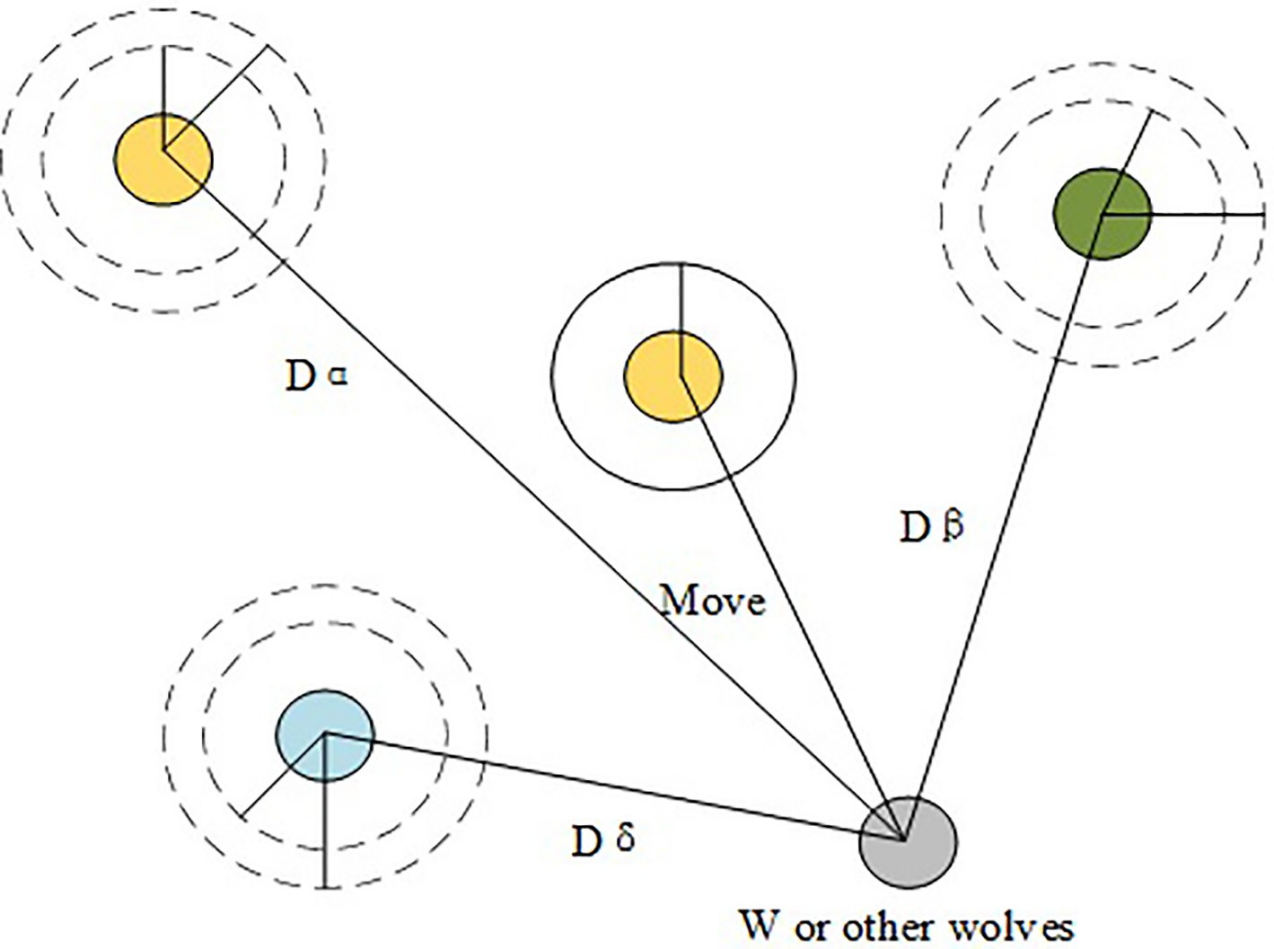
Structure of GWO.

GWO is a new meta-heuristic optimization algorithm with fast convergence speed and high optimization accuracy. Hence, it has excellent reference value in the application. GWO algorithm simulates the social organization leadership mechanism of the grey wolf packs. The group hunting behaviors, including searching for prey, surrounding prey, and hunting, can help obtain the optimal solution position via continuous iterative optimization [39]. The detailed principles are shown in Fig 5. There are three superior search individuals in the wolf packs, which are jointly responsible for specifying the movement direction of the inferior ω. Then, ω feeds back the information to the superior search individuals. Once the maximum number of iterations is met, the position of α is the optimal solution, the position of β is the sub-optimal solution, and the position of δ is the sub-sub-optimal solution [40]. GWO algorithm imitates the behaviors of wolves via three steps: surrounding, hunting, and attacking. The particular process is as follows:

1. Surrounding prey: the population will find the best route for hunting by surrounding the prey during optimizing. The following equations can determine the target position and the optimal population position in the surrounding phase:
  $$\vec{D}=|\vec{C}\cdot {\vec{X}}_{p}(t)-\vec{X}(t)|$$ (15)
  $$\vec{X}(t+1)={\vec{X}}_{p}(t)-\vec{A}\cdot \vec{D}$$ (16)
  In (15) and (16), t represents the current iteration number, $\vec{A}\cdot \vec{D}$ is the coefficient vector, ${\vec{X}}_{p}$ is the optimal target vector (the position of the prey), $\vec{X}(t)$ is the current position vector of a searching individual, and $\vec{X}(t+1)$ is the next moving direction vector. $\vec{A}$ and $\vec{C}$ can be represented by:
  $$\vec{\alpha }(t)=2(1-t/M)$$ (17)
  $$\vec{A}=2\vec{\alpha }\cdot {\vec{r}}_{1}-\vec{\alpha }$$ (18)
  $$\vec{C}=2{\vec{r}}_{2}$$ (19)
  In (17)–(19), M is the maximum number of iterations, $\vec{\alpha }$ decreases linearly to 0 as the number of iterations t increases, ${\vec{r}}_{1}$ and ${\vec{r}}_{2}$ are random vectors between [0,1]. Therefore, the points around the optimal solution are searched by adjusting the size of the coefficient vector $\vec{A}$ and $\vec{C}$. Furthermore, the local optimization ability of the algorithm is guaranteed. The optimization population can find all the offensive target paths while ensuring the algorithm’s global searchability.
2. Hunting and attacking: when hunting and attacking prey, according to the signal sent by *α*, *β*, *δ*, *ω* will move and determine whether it is close to the target or far away. This process can be expressed as:
  $$\{\begin{array}{l} \vec{D_{\alpha }}=|{\vec{C}}_{1}\cdot {\vec{X}}_{\alpha }-\vec{X}|\\ \vec{D_{\beta }}=|{\vec{C}}_{2}\cdot {\vec{X}}_{\beta }-\vec{X}|\\ \vec{D_{\delta }}=|{\vec{C}}_{3}\cdot {\vec{X}}_{\delta }-\vec{X}| \end{array}$$ (20)
  $$\{\begin{array}{l} \vec{X_{1}}={\vec{X}}_{\alpha }-\vec{A_{1}}\vec{D_{\alpha }}\\ \vec{X_{2}}={\vec{X}}_{\beta }-\vec{A_{2}}\vec{D_{\beta }}\\ \vec{X_{3}}={\vec{X}}_{\delta }-\vec{A_{3}}\vec{D_{\delta }} \end{array}$$ (21)
  $$\vec{X}(t+1)=(\vec{X_{1}}+\vec{X_{2}}+\vec{X_{3}})/3$$ (22)

In (20), (21), and (22), $\vec{D_{\alpha }},\vec{D_{\beta }},\vec{D_{\delta }}$ respectively, represents the direction vector between *α*, *β*, *δ* and *ω*, and $\vec{X_{1}},\vec{X_{2}},\vec{X_{3}}$ respectively represents the direction vector that *α*, *β*, *δ* determines the next move. GWO algorithm realizes the modeling of the entire process of iterative optimization based on the wolves’ hierarchical division of labor system and wolf packs’ hunting behaviors. GWO algorithm is applied to the parameter optimization of the FD and recognition network of the cutting control system, optimizing the parameters *c* and *g* of the SVM training network, thereby improving the accuracy and efficiency of fault classification and recognition.

### 3.3 GWO-SVM-based fault detection model

**Fig 6:**
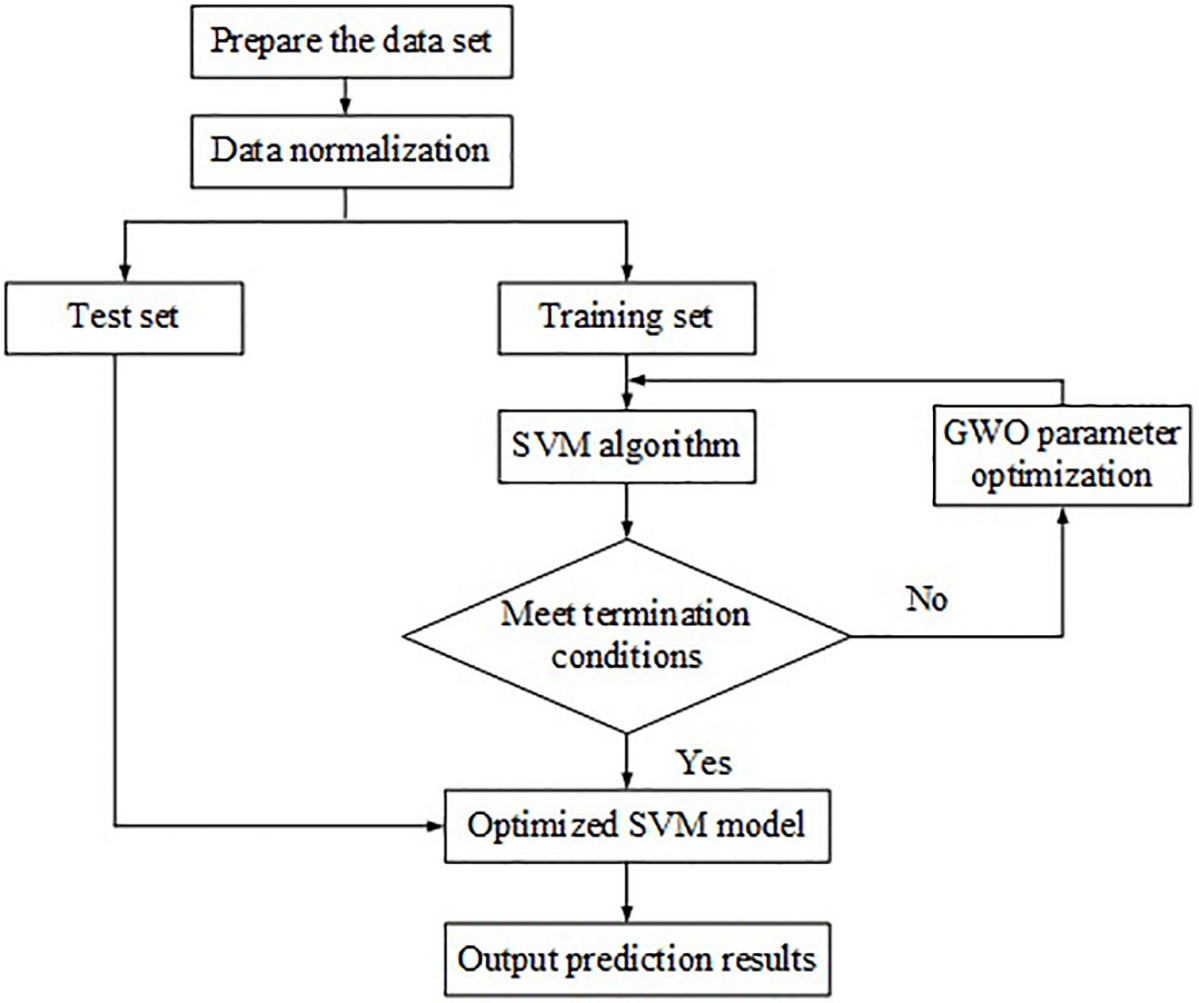
Automatic detection and recognition model of cutting control faults based on GWO-SVM algorithm.

An FD model is proposed based on GWO-SVM. The model’s core is optimizing the penalty coefficient *c* and the kernel function radius *g* of the SVM through the GWO algorithm. The optimal combination of *c* and are chosen to improve the classification accuracy and speed of SVM. The structure of the GWO algorithm is simple and easy to understand, which can be realized by setting a few parameters. GWO algorithm has a significant advantage in finding the optimal solution of SVM. The particular fault identification and the prediction model is shown in Fig 6. First, a dataset is prepared. After data normalization, the dataset is divided into a test set and a training set. After SVM processes the training set, it builds a fault prediction model based on the initial *c* and *g*, in an effort to minimize the error rate. Second, the positions are respectively updated according to the relations among the objective function’s values and the objective functions of α, β, and δ wolves. The positions are then divided into different levels, i.e., α, β, and δ, according to the fitness value. New *X*_*α*_, *X*_*β*_, and *X*_*δ*_ are determined according to the updated optimal objective function values. Finally, the GWO algorithm optimizes the data parameters that do not meet the requirements, and the best parameters *c* and *g* are utilized for constructing the prediction model, predicting the unknown data sample, and analyzing the test results.

### 3.4 Model parameters and training

1. Model parameter determination: the AlexNet model is chosen as the feature extraction model. AlexNet includes five convolutional layers, three fully-connected layers, and a Softmax layer at the bottom. A Local Response Normalization (LRN) layer comes after the first and second convolutional layers. The largest convergence layer appears in the two LRN layers and the last convolutional layer. The Rectified Linear Unit (ReLU) activation function is used at the end of each layer. Each state’s data is converted into a spectrogram. The trained AlexNet model is adopted to extract the features of the signal scale spectrogram. The feature extraction layer is *fc7*, and the number of iterations is 200. The AlexNet model can directly mine fault features from the original data, possessing a more vital ability to process high-dimensional and nonlinear data. Hence, it can clearly distinguish the health status of leather cutting and provide better support for the classification model. The performance parameters for different models are shown in Table 1.
2. Model training: the cross-validation method is adopted. Generally, the data are divided into three sets randomly: the training set, the validation set, and the test set. The training set is utilized for model training. The validation set is applied to evaluate the prediction of the model and select the model parameters. Finally, the models are run on the test set to decide which model to use and the corresponding parameters. The experimental data are the operating data of the control system of a leather cutting company for 5 years. The training set, test set, and verification set account for 7:2:1 [41]. First, the operational data are extracted and scaled to fall into a small interval. The unit limit of the data is removed, and the data are converted into a dimensionless pure value, convenient for indicators of different units or magnitudes to be compared and weighted. Then the data are normalized, and the original data are linearly transformed so that the result falls into the [0,1] interval, where max is the maximum value of the sample data, and min is the minimum value of the sample data. The training sample set trains the SVM, the test sample set tests the trained SVM, and whether the diagnosis is accurate. Finally, the failure can be automatically detected by the SVM network that reaches the requirements. In the experiments, 500 normal samples, 1,000 inner ring failure samples, 1,000 outer ring failure samples, and 1,000 rolling element failure samples are selected to form the training set, totaling 3,500 samples. In the meantime, 1,000 samples are selected to form the test sample set, 500 samples are the validation set. All experiments perform 10 times of diagnosis, each failure analysis is independently completed. Each diagnosis repeats three experiments, and the average value is taken as the final result [42].

**Table 1:** Performance parameters for different models.

Model/Parameter	SVM	CV-SVM	GA-SVM	PSO-SVM	GWO-SVM	CNN-SVM	GA	PSO
Penalty coefficient c	10^2	10^2	10^2	10^2	10^2	10^2	10^2	10^2
Kernel function	Linear kernel	Linear kernel	Linear kernel	Linear kernel	Linear kernel	Linear kernel	Linear kernel	Linear kernel
Kernel function radius g	0.01	0.01	0.01	0.01	0.01	0.01	0.01	0.01
Group size	-	20~100	20~100	20~100	20~100	20~100	20~100	20~100
Number of iterations	200	200	200	200	200	200	200	200
Learning factors C1 and C2	-	-	-	2	-	-	2	-
Convolution kernel size	-	-	-	-	5x3	3x3,5x5	-	-
Number of convolution kernels	-	-	-	-	-	2	-	-
Crossover rate	-	-	0.5	-	-	-	0.5	-
Mutation rate	-	-	0.06	-	-	-	0.06	-
Inertia weight	-	-	-	0.9	-	-	-	0.9

### 3.5 Model simulation and performance text

1. Model simulation: the hardware is: Intel(R) Core(TM) i5-8300H 2.300 GHz, 8 GB internal memory, 64-bit operating system; the software is MATLAB R2019a. The details are shown in Table 2.
2. Performance test: the proposed GWO-SVM model is compared with traditional SVM, OpenCV (CV), GA [43], PSO [44], Convolution Neural Network (CNN), and GWO. The optimal algorithm model optimizes the penalty coefficient c of the SVM and the radius g of the kernel function, thereby comparing the fitness curves of the obtained classification models for comparative experiments. Accuracy (Acc), Precision (Pre), Recall (Rec), and F1 are criteria for evaluating the model performance [45]. The details are shown in the following equations:
  $$Acc=\frac{P_{1}+P_{2}}{P_{1}+P_{2}+P_{3}+P_{4}}$$ (23)
  $$\text{Pr}\;e=\frac{P_{1}}{P_{1}+P_{2}}$$ (24)
  $$\text{Re}\;\text{call}=\frac{P_{2}}{P_{2}+P_{4}}$$ (25)
  $$F_{1}=\frac{2*\text{Pr}\;e*\text{Re}\;c}{\text{Pr}\;e+\text{Re}\;c}$$ (26)
  In (23)–(26), *P*_*1*_ is the number of faults correctly identified by this model, *P*_*2*_ is the number of non-faults correctly identified by this model, *P*_*3*_ is the number of unrecognized faults, and *P*_*4*_ is the number of unrecognized non-faults.
3. Statistical analysis: The data obtained are processed using SPSS 24.0 (SPSS Inc., Chicago, Illinois, USA) on the Windows platform. Continuous variables with normal distribution are expressed as mean ± standard deviation (SD), and non-normal variables are reported as median (interquartile range). The average of two continuous normally distributed variables is compared through the independent sample T-test, and it is considered significant when P<0.05. Data are visualized using Origin 2019 64 Bit and Visio 2013 [46].

**Table 2:** Experimental environment.

Simulation Environment	Parameters
Computer system version	Ubuntu 14.04 OS
GPU graphics card model	Intel(R) Core(TM) i5-8300H
Video memory	11G
RAM	16G
Programming language	Python 3.6.3

## 4. Results and analyses

### 4.1 Comparative analysis of algorithm performance

Tables 3–6 demonstrate the performance comparison of different algorithms under different detection number sets. In terms of Pre and Rec of fault prediction, the GWO-SVM model presents the best performance, whose Pre can reach 92.69%. However, the Acc of the GWO-SVM model is not excellent. A possible reason is that the Acc indicator belongs to mixed calculation; for the model with less number of tests, the performance difference is inferior. The performance of CNN-SVM ranks second, with the highest Pre reaching 87.24%. Compared with GA, PSO, and CV, the CNN network has multi-threaded data analysis capabilities; as the number of data increases, the model Acc is continuously improving. The above results prove that the proposed GWO-SVM algorithm shows better performance in fault prediction.

**Table 3:** Accuracy results of different algorithms under the detection number sets.

ACC (%)	SVM	CV-SVM	GA-SVM	PSO-SVM	GWO-SVM	CNN-SVM
1	0.726782	0.871621	0.884682	0.885907	0.927351	0.873309
2	0.785154	0.884181	0.880707	0.889129	0.929161	0.882132
3	0.794099	0.871287	0.883244	0.883678	0.873954	0.870088
4	0.758205	0.882511	0.884272	0.872653	0.906775	0.885144
5	0.823153	0.873959	0.885622	0.88482	0.884799	0.883401
6	0.786202	0.88515	0.877553	0.875782	0.871252	0.878924
7	0.751107	0.868417	0.871228	0.88173	0.904868	0.882008
8	0.777529	0.888537	0.880383	0.883542	0.877969	0.884103
9	0.81695	0.874892	0.88404	0.883543	0.880639	0.88228
10	0.82924	0.866494	0.871463	0.873772	0.915982	0.882608
Average value	0.784842	0.876705	0.880319	0.881455	0.897275	0.8804

**Table 4:** Precision results of different algorithms under the detection number sets.

Pre (%)	SVM	CV-SVM	GA-SVM	PSO-SVM	GWO-SVM	CNN-SVM
1	0.808324	0.866346	0.874321	0.860915	0.902109	0.863094
2	0.801351	0.864088	0.866996	0.864134	0.929002	0.872406
3	0.785509	0.876461	0.87114	0.86162	0.91477	0.862436
4	0.805348	0.86477	0.8637	0.873317	0.922296	0.870137
5	0.789317	0.877593	0.874373	0.861823	0.926976	0.878288
6	0.766259	0.878374	0.867307	0.879116	0.925773	0.867187
7	0.794223	0.871204	0.867427	0.861411	0.91091	0.864729
8	0.76695	0.86552	0.864569	0.879743	0.916905	0.860504
9	0.784628	0.871181	0.871995	0.871438	0.902054	0.862811
10	0.795072	0.873138	0.875402	0.864881	0.923252	0.872001
Average value	0.789698	0.870867	0.869723	0.86784	0.917405	0.867359

**Table 5:** Recall results of different algorithms under the detection number sets.

Rec (%)	SVM	CV-SVM	GA-SVM	PSO-SVM	GWO-SVM	CNN-SVM
1	0.793634	0.870106	0.875302	0.86952	0.925487	0.863733
2	0.755136	0.863404	0.872078	0.877887	0.927678	0.864295
3	0.734558	0.872757	0.869155	0.872547	0.903599	0.87424
4	0.768872	0.867445	0.872721	0.876091	0.914801	0.873173
5	0.824158	0.866017	0.871584	0.872734	0.91576	0.877635
6	0.812635	0.870826	0.86746	0.870054	0.906597	0.866932
7	0.780835	0.862633	0.86219	0.863347	0.911978	0.875639
8	0.758155	0.877469	0.864685	0.87726	0.926811	0.876715
9	0.827515	0.86042	0.871679	0.878606	0.904712	0.861555
10	0.793496	0.877836	0.872376	0.874123	0.916074	0.868725
Average value	0.784899	0.868891	0.869923	0.873217	0.91535	0.870264

**Table 6:** F1-score results of different algorithms under the detection number sets.

F1-score	SVM	CV-SVM	GA-SVM	PSO-SVM	GWO-SVM	CNN-SVM
1	0.800911	0.868222	0.874811	0.865196	0.913649	0.863413
2	0.777558	0.863746	0.869529	0.870956	0.92834	0.868332
3	0.75918	0.874605	0.870146	0.867049	0.90915	0.868298
4	0.786687	0.866106	0.868187	0.874702	0.918533	0.871653
5	0.806361	0.871766	0.872976	0.867244	0.921334	0.877962
6	0.788766	0.874583	0.867384	0.874561	0.916084	0.867059
7	0.787472	0.866897	0.864801	0.862378	0.911443	0.87015
8	0.762527	0.871454	0.864627	0.8785	0.921831	0.868534
9	0.805501	0.865767	0.871837	0.875008	0.903381	0.862183
10	0.794283	0.875481	0.873886	0.869477	0.919649	0.87036
Average value	0.786925	0.869863	0.869818	0.870507	0.916339	0.868794

### 4.2 Determination of optimal parameters

Table 7 indicates the result of optimizing different *c* and *g* values under the GWO-SVM model. When the *c* value is 25, the Acc of the model reaches 96%, and the classification requires at least 337s. When the *g* value is 0.2, the model Acc reaches 95%, and the classification costs 352s. According to the above results, the subsequent experiments are conducted under the conditions of *c* = 25 and *g* = 0.2.

**Table 7:** Parameter optimization results under the GWO-SVM model.

Parameters	Change value	ACC (%)	Time (s)
c	10	0.85	435
	15	0.86	424
	20	0.9	406
	25	0.96	337
	30	0.95	387
	35	0.93	392
	40	0.92	410
g	0.01	0.93	403
	0.02	0.95	352
	0.03	0.89	395
	0.04	0.86	410
	0.05	0.88	424
	0.06	0.85	436
	0.07	0.8	445

### 4.3 Comparison with traditional models

As shown in Tables 8 and 9, the Case Western Reserve University electrical engineering experimental dataset is utilized to test the traditional model’s fault handling results and the proposed GWO-SVM under different datasets. In terms of model Acc in fault prediction, the GWO-SVM model is significantly better than other traditional models, with the highest average Acc reaching 99.24%, which is 15.6% higher than that of the traditional models. The number of different fault predictions is compared with the time to obtain specific model processing efficiency. The average processing efficiency of the GWO-SVM model is 0.8667, while that of the traditional models is 0.2864; the former is 66.96% higher than the latter. The above results prove the effectiveness of the proposed GWO-SVM model.

**Table 8:** Accuracy results compared with traditional models.

	Traditional method (%)	GWO-SVM (%)
100	0.8635	0.9836
200	0.8534	0.9924
300	0.8247	0.9815
400	0.8351	0.9935
500	0.8016	0.9814
600	0.8219	0.9925
Average value	0.833367	0.987483

**Table 9:** Fault handling efficiency results compared with traditional models.

	Traditional method (times/min)	GWO-SVM (times/min)
100	0.18978	0.292738
200	0.299965	0.560678
300	0.299528	0.756941
400	0.326211	0.991022
500	0.350044	1.191019
600	0.253022	1.407801
Average value	0.286425	0.8667

### 4.4 Model performance verification and computational complexity

The Wilcoxon method tests the model. The results are summarized in Tables 10–14. The obtained results are consistent with the results of Section 4.1 above. There are significant differences between the SVM algorithm and other algorithms. In terms of the model accuracy, there is no significant difference between the GWO-SVM and CNN-SVM algorithms (p >0.05). According to accuracy, recall, and F1 results, the proposed algorithm is significantly better than other algorithms. There are significant differences between the proposed algorithm and other algorithms (p<0.001). The traditional algorithms and the proposed algorithm are tested as well, revealing significant differences. Hence, the proposed algorithm has obvious advantages in performance.

**Table 10:** Computational complexity test results for accuracy of different models.

ACC (%)	SVM	CV-SVM	GA-SVM	PSO-SVM	GWO-SVM	CNN-SVM
1	0.72678	0.87162	0.88468	0.88591	0.92735	0.87331
2	0.78515	0.88418	0.88071	0.88913	0.92916	0.88213
3	0.7941	0.87129	0.88324	0.88368	0.87395	0.87009
4	0.75821	0.88251	0.88427	0.87265	0.90678	0.88514
5	0.82315	0.87396	0.88562	0.88482	0.8848	0.8834
6	0.7862	0.88515	0.87755	0.87578	0.87125	0.87892
7	0.75111	0.86842	0.87123	0.88173	0.90487	0.88201
8	0.77753	0.88854	0.88038	0.88354	0.87797	0.8841
9	0.81695	0.87489	0.88404	0.88354	0.88064	0.88228
10	0.82924	0.86649	0.87146	0.87377	0.91598	0.88261
Average value	0.784842	0.876705	0.880318	0.881455	0.897275	0.880399

**Table 11:** Computational complexity test results for precision of different models.

Pre (%)	SVM	CV-SVM	GA-SVM	PSO-SVM	GWO-SVM	CNN-SVM
1	0.80832	0.86635	0.87432	0.86092	0.90211	0.86309
2	0.80135	0.86409	0.867	0.86413	0.929	0.87241
3	0.78551	0.87646	0.87114	0.86162	0.91477	0.86244
4	0.80535	0.86477	0.8637	0.87332	0.9223	0.87014
5	0.78932	0.87759	0.87437	0.86182	0.92698	0.87829
6	0.76626	0.87837	0.86731	0.87912	0.92577	0.86719
7	0.79422	0.8712	0.86743	0.86141	0.91091	0.86473
8	0.76695	0.86552	0.86457	0.87974	0.91691	0.8605
9	0.78463	0.87118	0.872	0.87144	0.90205	0.86281
10	0.79507	0.87314	0.8754	0.86488	0.92325	0.872
Average value	0.789698	0.870867	0.869724	0.86784	0.917405	0.86736

**Table 12:** Computational complexity test results for recall of different models.

Rec (%)	SVM	CV-SVM	GA-SVM	PSO-SVM	GWO-SVM	CNN-SVM
1	0.79363	0.87011	0.8753	0.86952	0.92549	0.86373
2	0.75514	0.8634	0.87208	0.87789	0.92768	0.8643
3	0.73456	0.87276	0.86916	0.87255	0.9036	0.87424
4	0.76887	0.86745	0.87272	0.87609	0.9148	0.87317
5	0.82416	0.86602	0.87158	0.87273	0.91576	0.87764
6	0.81264	0.87083	0.86746	0.87005	0.9066	0.86693
7	0.78084	0.86263	0.86219	0.86335	0.91198	0.87564
8	0.75816	0.87747	0.86469	0.87726	0.92681	0.87672
9	0.82752	0.86042	0.87168	0.87861	0.90471	0.86156
10	0.7935	0.87784	0.87238	0.87412	0.91607	0.86873
Average value	0.784902	0.868893	0.869924	0.873217	0.91535	0.870266

**Table 13:** Computational complexity test results for F1-score of different models.

F1-score	SVM	CV-SVM	GA-SVM	PSO-SVM	GWO-SVM	CNN-SVM
1	0.80091	0.86822	0.87481	0.8652	0.91365	0.86341
2	0.77756	0.86375	0.86953	0.87096	0.92834	0.86833
3	0.75918	0.87461	0.87015	0.86705	0.90915	0.8683
4	0.78669	0.86611	0.86819	0.8747	0.91853	0.87165
5	0.80636	0.87177	0.87298	0.86724	0.92133	0.87796
6	0.78877	0.87458	0.86738	0.87456	0.91608	0.86706
7	0.79428	0.87548	0.87389	0.86948	0.91965	0.87036
8	0.76253	0.87145	0.86463	0.8785	0.92183	0.86853
9	0.8055	0.86577	0.87184	0.87501	0.90338	0.86218
10	0.79428	0.87548	0.87389	0.86948	0.91965	0.87036
Average value	0.787606	0.870722	0.870729	0.871218	0.917159	0.868814

**Table 14:** Fault handling efficiency compared with traditional models.

	Traditional method (Times/min)	GWO-SVM (Times/min)
100	0.8635	0.9836
200	0.8534	0.9924
300	0.8247	0.9815
400	0.8351	0.9935
500	0.8016	0.9814
600	0.8219	0.9925
Average value	0.833367	0.987483

The model proposed is compared with the state of the art methods. Its complexity is expressed as the algorithm processing efficiency per unit time. The results are shown in Table 15. Under the fixed experimental conditions, the traditional feature extraction methods and deep feature extraction methods are compared. Feature extraction (CNN) by comparing wavelet packet feature extraction and deep learning shows that using AlexNet for deep feature extraction, the obtained features are more obvious, making the classification effect more excellent. Besides, the optimization time is compared as well. Using deep learning for feature extraction requires less optimization time than traditional methods. Moreover, whether it is traditional feature extraction or deep extraction, the GWO-SVM model is far superior to the PSO-SVM model and the GA-SVM model in terms of training time and testing time, which improves the speed of model classification. In terms of the classification accuracy, the features extracted by the deep learning method are more precise and effective, so that more useful features can be input into the classifier, making the classification accuracy higher. Also, the classification accuracy of the GWO-SVM model is higher than the PSO-SVM model and the GA-SVM model. According to Table 10, the recognition rate of the GWO-SVM model is greatly improved with the increase in the number of diagnoses. The optimization time and diagnostic accuracy are the key factors to measure the diagnosis model. Compared with the state of the art algorithms, the performance of the proposed algorithm model in complexity is 0.29439, which is better than other models. Therefore, the GWO-SVM model has strong practicability in rolling machinery FD.

**Table 15:** Failure processing efficiency results compared with other models.

	Accuracy/%	Classification time/s	Algorithm complexity
Wavelet packet-GA-SVM [19]	83.25	2415.94	0.034458637
Wavelet packet-PSO-SVM [20]	89.25	3860.78	0.02311709
Wavelet packet-GWO-SVM [21]	89.25	1249.86	0.071407998
CNN-GA-SVM [22]	97.29	435.13	0.223588353
CNN-PSO-SVM [23]	99.26	681.00	0.145756241
GWO-SVM	99.27	337.2	0.294395018

## 5. Discussion

An FD method for leather cutting is proposed based on deep learning feature extraction and GWO-SVM. The signal features can be better obtained by converting the signal into a scale spectrum and using the SVM network for feature extraction. The GWO algorithm is employed to optimize SVM; in this way, the adjustment parameters are reduced, the optimization speed is fast, and the classification accuracy is high. After parameter optimization, this method can significantly improve the accuracy of FD. This is also verified in the study of Fu et al. (2019), in which they proposed an FD method for rotating machinery based on the blind parameter identification of the MAR model and the mutation hybrid GWO-SCA optimization; the actual application and comparative analysis proved the effectiveness and superiority of this method [47]. The SVM algorithm is based on the statistical learning theory and the principle of structural risk minimization. It minimizes the confidence risk by fixing the empirical risk and maps the input space to the high-dimensional inner product space, effectively avoiding the “dimensionality disaster.” It has significant advantages in solving small sample sets and nonlinear high-dimensional pattern recognition problems, which has received widespread attention in the field of FD. The simulation results also show that compared with the traditional algorithms, the average accuracy rate of the proposed algorithm is increased by 15.62%, which has also been verified in previous reports. Yan and Jia (2018) proposed an optimization-based support Multi-domain feature fault classification algorithm of SVM; this algorithm included three stages: multi-domain feature extraction, feature selection, and feature recognition; finally, the experimental analysis found that the proposed method could achieve higher diagnosis accuracy under different working conditions and was better than the traditional methods mentioned above and published in other literature [48].

The structure of the GWO algorithm is simple and easy to understand, which can be realized by setting a few parameters. GWO algorithm has a significant advantage in finding the optimal SVM solution, which is also proved in the above simulation results. Using deep learning for feature extraction requires less optimization time than traditional feature extraction. Moreover, whether it is traditional feature extraction or deep extraction, the speed of the GWO-SVM model is far superior to the PSO-SVM model and the GA-SVM model in terms of training time and test time, which improves the speed of model classification. In terms of classification accuracy, the features extracted by deep learning features are more apparent and significant so that more useful features can be input into the classifier, making the classification accuracy higher. Besides, the classification accuracy of the GWO-SVM model is higher than the PSO-SVM model and the GA-SVM model. As the number of diagnoses increases, the recognition rate of the GWO-SVM model is greatly improved. The optimization time and diagnostic accuracy are the key factors to measure the diagnosis model. Hence, the GWO-SVM model has strong practicability in the FD of leather cutting. Dong et al. (2019) combined the advantages of TSMWPE and proposed an intelligent FD method for rolling bearings combined with GWO-SVM. The FD method was applied to the experimental data analysis of two rolling bearings. The results showed that the method could accurately diagnose the fault category and severity of rolling bearings, and the corresponding recognition rate was higher than the current comparison method [49], which is consistent with the above results. On the one hand, nature, degree, class, location, cause, and development trend of the fault can be determined, thereby providing an accurate reference for the following forecasting, control, adjustment, and maintenance. On the other hand, experiences can be accumulated for future FD of the cutting machines, and appropriate solutions can be chosen for different fault types and degrees.

Feature extraction using deep learning requires less optimization time than traditional feature extraction. Moreover, whether it is traditional feature extraction or deep extraction, the speed of the GWO-SVM model is far superior to the PSO-SVM model and the GA-SVM model in terms of training time and test time, which improves the speed of model classification. In terms of classification accuracy, the features extracted by deep learning features are more apparent and significant so that more useful features can be input into the classifier, making the classification accuracy higher. Besides, the classification accuracy of the GWO-SVM model is higher than the PSO-SVM model and the GA-SVM model. As the number of diagnoses increases, the recognition rate of the GWO-SVM model is significantly improved. The optimization time and diagnostic accuracy are the key factors to measure the diagnosis model. Hence, the GWO-SVM model has strong practicability in the FD of leather cutting. When the Gaussian kernel’s radius is minimal, over-fitting will occur due to the classifier’s over-reliance on training samples, resulting in poor classification results. As Gaussian kernel’s parameters increase, the algorithm’s performance gradually improves. Once the parameter reaches a particular value, the classifier’s learning ability begins to deteriorate gradually, and the error rate also increases. The reason is that the proposed algorithm is a down-sampling algorithm, and the selected samples are representative. Therefore, SVM and GWO algorithms have particular advantages and can well exert these advantages in dealing with faults.

## 6. Conclusions

Problems in the current leather cutting system are analyzed deeply. A fault detection model is constructed according to the principles of SVM. Then, the GWO algorithm parameters that influence the recognition effect, i.e., the penalty factor parameter *c* and the kernel function parameter *g*, are optimized. The model is trained by 5-year operational data. Afterward, it can learn and recognize the feature vectors that characterize the fault mode. Finally, the experimental results prove that the hybrid FD model using the GWO-SVM classification network has a better recognition effect. Compared with other models, the GWO-SVM model has higher cutting accuracy and more straightforward system operation, which can provide a theoretical basis for the process improvement of leather cutting enterprises. Although the model’s accuracy is high, several problems are found, and some methods need improving. First, the accuracy of fault pattern recognition is connected to selecting feature vectors and closely correlated to the training network’s parameter settings. In the future, the method of network identification optimization can be improved to analyze and mine the data’s internal structure, thereby improving FD’s identification efficiency and accuracy. Second, the operating condition database of all cutting sensing systems can be established. The control signals are collected, analyzed, judged, and diagnosed in real-time using the computer and other software systems to monitor the cutting machines. These two aspects will be explored in-depth to improve the fault detection models of cutting machines in the future.

## Supporting information

S1 Data (ZIP)
